# Aberrant NRP-1 expression serves as predicator of metastatic endometrial and lung cancers

**DOI:** 10.18632/oncotarget.6699

**Published:** 2015-12-20

**Authors:** Imoh S. Okon, Ye Ding, Kathleen A. Coughlan, Qiongxin Wang, Ping Song, Doris M. Benbrook, Ming-Hui Zou

**Affiliations:** ^1^ Center for Molecular and Translational Medicine, Georgia State University, Atlanta, GA 30302, USA; ^2^ Section of Molecular Medicine, Oklahoma City, OK 73104, USA; ^3^ Department of Obstetrics and Gynecology, University of Oklahoma Health Sciences Center (OUHSC), Oklahoma City, OK 73104, USA

**Keywords:** NRP-1, NEDD9, LKB1, tumor metastases, endometrial and lung cancer

## Abstract

Neuropilin-1 (NRP-1) has emerged as an important driver of tumor-promoting phenotypes of human malignancies. However, incomplete knowledge exists as to how this single-pass transmembrane receptor mediates pleiotropic tumor-promoting functions. The purpose of this study was to evaluate NRP-1 expression and metastatic properties in 94 endometrial cancer and matching serum specimens and in a lung cancer cell line. We found that NRP-1 expression significantly correlated with increased tumoral expression of vascular endothelial growth factor 2 (VEGFR2) and serum levels of hepatocyte growth factor (HGF) and cell growth-stimulating factor (C-GSF). Tumoral NRP-1 also was positively associated with expression of NEDD9, a pro-metastatic protein. In the highly metastatic lung cancer cell line (H1792), stable LKB1 depletion caused increased migration *in vitro* and accentuated NRP-1 and NEDD9 expression *in vivo*. Our findings demonstrate that perturbed expression of these targets correlate with metastatic potential of endometrial and lung tumors, providing clinically-relevant biomarker applications for diagnostic and therapeutic targeting.

## INTRODUCTION

Mortality rates arising from endometrial and lung cancers, like most other cancer-types is strongly due to metastasis which involves the spread of malignant cells from primary tissues to secondary sites. Specificity, and drug targeting of metastatic tumors is difficult to achieve, contributing to poor response and eventual drug resistance. To this end, early identification of genetic markers as predicators of tumor metastatic potential may allow early diagnoses and precision-guided therapeutic interventions. This study presents findings from clinical specimens that were obtained from endometrial cancer patients with a bid to understanding predicators of metastatic property which directly impact prognoses. Brain metastases in lung cancer patients and lymph node metastases in endometrial cancer patients are significant predictors of poor patient survival [1, 2].

Tumor angiogenesis, which is an important component in the development of metastases, has been targeted by inhibition of pro-angiogenic factors, including vascular endothelial growth factor (VEGF) and tyrosine kinases, in both lung and endometrial cancer [3]. Bevacizumab, a humanized monoclonal antibody to VEGF, is FDA approved in combination with chemotherapy for treatment of advanced non-small cell lung cancer (NSCLC) and several small molecule tyrosine kinase inhibitors, including, Sunitinib, Cediranib and Vatalanib, are currently being investigated in NSCLC clinical trials [4]. Phase II trials of bevacizumab or Sunitinib demonstrated promising activities in advanced or recurrent endometrial cancer [5, 6]. While these results are promising and provide short-term benefits, the eventual development of resistance indicate that greater understanding of the roles of angiogenic factors in metastases are needed [7, 8].

Given well-established pleiotropic tumor-promoting functions, and canonical pro-angiogenic roles that support survival of malignant cells, neuropilin-1 (NRP-1) was an attractive target to investigate. NRP-1 is a 120–130 kDa, type I transmembrane protein that acts as a co-receptor to the semaphorins and VEGF families. NRP-1 has been strongly associated with additional growth-factors [9–11], suggesting a positive feedback loop that allows the induction of cancer-promoting properties through the respective receptors. While NRP-1 is a non-tyrosine kinase receptor, it supports co-receptor signaling functions of several tyrosine kinase receptors, including epidermal growth factor receptor (EGFR) and VEGF receptor 2 (VEGFR2) [9]. Due to well-established links with VEGFR2, the single-pass NRP-1 receptor has been strongly associated with tumor angiogenesis [12, 13]. Prenatal death of global *NRP-1*^−/−^ mice resulted from defective neuronal patterning and vessel formation [14–17]. Unsurprisingly, NRP-1 is highly expressed in various human neoplasm and contribute to tumor growth, neovascularization and metastasis [14–22]. Aberrant NRP-1 expression is further linked to poor prognosis within human tumors [10, 23, 24].

Prominent NRP-1 roles in axonal guidance of developing nervous system is in agreement with reported metastatic property in malignant cells [18, 23], however, the expression profile and potential contributions of NRP-1 to endometrial cancer metastases remain unknown. While NRP-1 typically localizes at the cell surface, its transmembrane protein structure suggest potential interaction with several membrane-associated proteins that possess diverse functions, including the maintenance of cell membrane integrity. Therefore, defective NRP-1 trafficking or degradation that result in its aberrant expression and/or membrane accumulation may potentially impact cell-membrane integrity, and hence tumor metastases.

Mutations of Liver Kinase B1 (*LKB1*) gene typically include point mutations and large deletions, and have been identified in several human malignancies, especially lung cancer [25, 26]. LKB1 loss-of-function mutations have been reported in association with tumor-enhancing phenotypes, suggesting that LKB1 is a regulator of tumor inhibitory processes. LKB1 possess several downstream substrates, including, AMP-activated protein kinase (AMPK), which is associated with sensing and modulation of cellular energy [27]. We have previously demonstrated abrogation of aberrant tyrosine kinase activity, as well as enhanced NRP-1 trafficking and degradation by LKB1 in lung cancer cells [9, 28]. In this study, we evaluated aberrant NRP-1 expression as an indicator of metastatic potential within endometrial and lung tumors. We further tested whether LKB1-mediated abrogation of NRP-1 correlated with reduced metastatic potential within cancer cells. Findings from the study highlight clinically relevant biomarkers with potential applications for the diagnoses and/or therapeutic targeting of tumors with metastatic potential.

## RESULTS

### Association of NRP-1 with tumor-enhancing cytokines in endometrial cancer specimens

**Figure 1:**
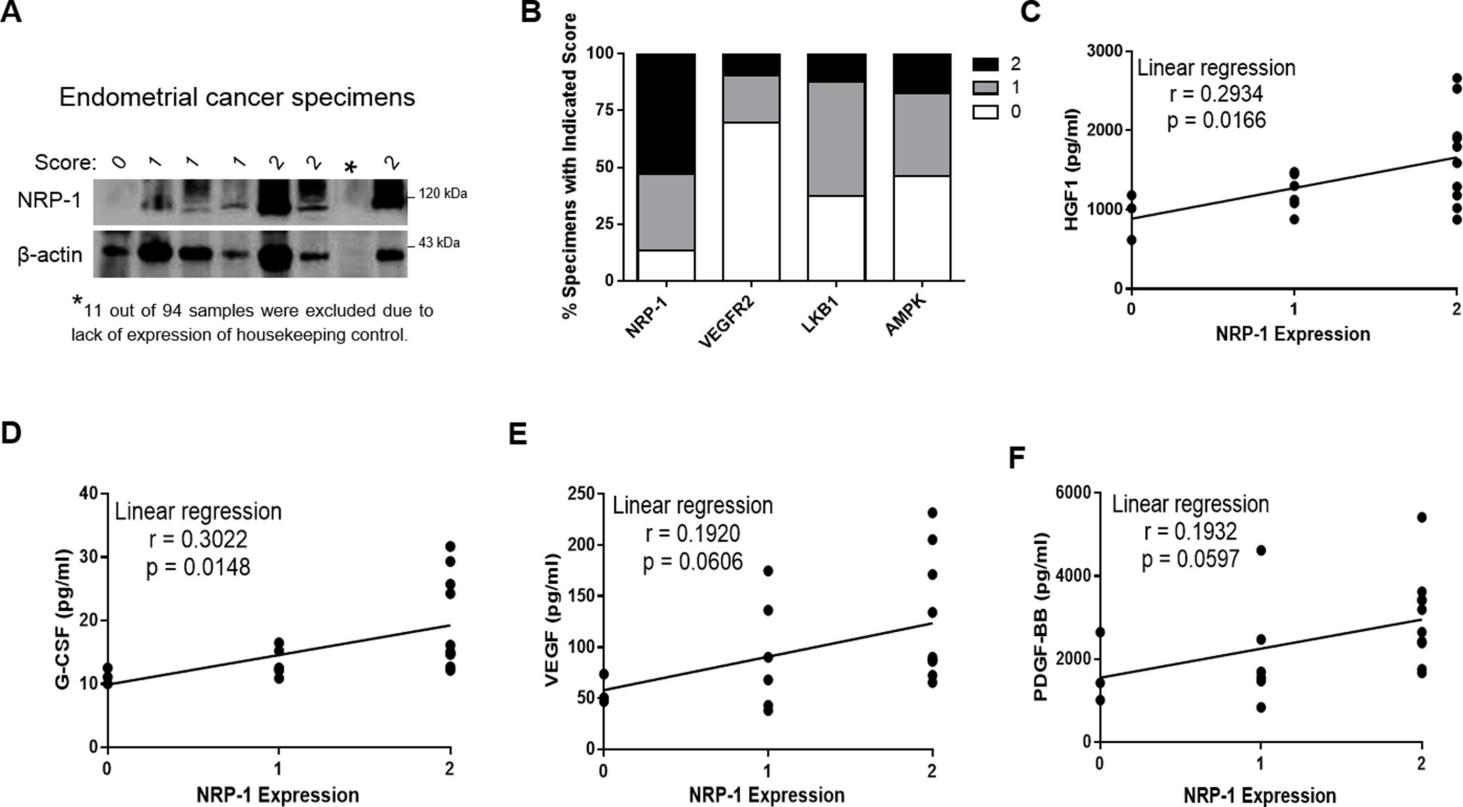
Association of NRP-1 with tumor-enhancing cytokines in endometrial cancer specimens (**A**) Example Western blots of NRP-1 expression in patient tumors. (**B**) Percentage of specimens exhibiting 0, 1 or 2 stain scores for the indicated biomarkers. Specific protein expression in clinical endometrial cancer specimens were normalized, and intensities scored as high (2), moderate (1) or negligible/undetected (0). (**C**–**F**) measurement of various tumor-enhancing cytokines by ELISA revealed significant HGF and G-CSF serum levels in patients with NRP-1 positive versus negative endometrial cancer using the Mann-Whitney test. Although VEGF and PDGF-BB showed a similar trend, the values were not significant.

Due to tumor functions associated with aberrant NRP-1 expression in various human malignancies, we began by screening endometrial cancer specimens for the expression of NRP-1 using Western blots of protein extracts from frozen specimens. Based on normalized intensities, expression levels were scored as high (2), moderate (1) or negligible/undetected (0). The tumor profile of our patient cohort is summarized in Table 1. Approximately 87% (72 of 83) of the specimens possessed moderate-to-high NRP-1 expression, while 13% (11 of 83) displayed negligible or undetected NRP-1 (Figure 1A and 1B). Majority of the specimens were from early stage cancer patients (Table 1), indicating that aberrant NRP-1 expression may represent an early event in endometrial cancer. NRP-1 mediates signals from multiple cytokines, and its aberrant expression can potentially impact the balance of circulating cytokines. Hence, we evaluated matched serum of the endometrial cancer tissue specimens using a multiplex kit that detects angiopoietin-2 (Ang-2), follistatin, granulocyte-colony stimulating factor (G-CSF), hepatocyte growth factor (HGF), interleukin-8 (IL-8), platelet-derived growth factor (PDGF)-BB, platelet endothelial cell adhesion molecule (PECAM-1) and VEGF. Higher levels of NRP-1 expression in the endometrial tumors were associated with increasing levels of circulating HGF and G-CSF in matching patient serum (*p* = 0.0166 and 0.0148 respectively; Figure 1C and 1D). Although VEGF and PDGF-BB showed similar trends, the *p* values did not achieve statistical significance (0.0606 and 0.0597 respectively; Figure 1E and 1F). Furthermore, associations between NRP-1 and the other cytokines were not observed (data not shown). Together, these data demonstrate robust NRP-1 expression in early-stage endometrial cancer, and shows positive associations of NRP-1 expression in tumors with circulating levels of various tumor-promoting cytokines.

**Table 1:** Endometrial cancer patient

Pathological stage		Pathological grade		
		1	2	3
I	Total	13	8	2
	*Average age	61.46 ± 3.96	62.75 ± 4.14	51.00 ± 16.00
II	Total	8	25	4
	*Average age	63.00 ± 2.34	69.72 ± 2.56	74.75 + 0.85
III	Total	3	18	2
	*Average age	71.00 ± 6.25	67.33 ± 2.82	83.50 ± 4.50

* Age at diagnosis

### Correlation of NRP-1, VEGFR2, LKB1 and AMPK expression in endometrial cancer specimens

We screened further the endometrial cancer specimens for biomarkers associated with the NRP-1 and/or LKB1 signaling pathway by Western blot analysis: VEGFR2, a co-receptor for NRP-1, which is strongly linked with tumor-promoting phenotypes on one hand, and AMPK, a downstream substrate of the candidate LKB1 tumor suppressor gene. Although NRP-1 was not expressed in the minority of specimens and VEGFR2 was not expressed in the majority of specimens, when their expression levels were compared within individual specimens, there was a significant positive correlation (Figure 1B and Table 2). This is consistent with reports that NRP-1 functions are associated with VEGFR2 [12, 13]. Although LKB1 and AMPK were also not expressed in the minority of specimens, their expression levels did not correlate with NRP-1 expression levels within the same tumors (Figure 1B and Table 2). LKB-1 expression positively correlated with AMPK and VEGFR2 expression, however AMPK and VEGFR2 expression did not correlate with each other (Table 2).

**Table 2:** Spearman correlation R and P values for associations between the indicated biomarkers

Spearman	NRP-1/ VEGFR2	NRP-1/LKB1	NRP-1/AMPK	LKB1/AMPK	LKB1/ VEGFR2	AMPK/ VEGFR2
r	0.2344	0.0977	0.1332	0.3226	0.2505	0.1065
P	*0.0319	0.3857	0.2357	*0.0033	*0.0241	0.344

### NRP-1 positively correlates with NEDD9 expression in endometrial cancer specimens

**Figure 2:**
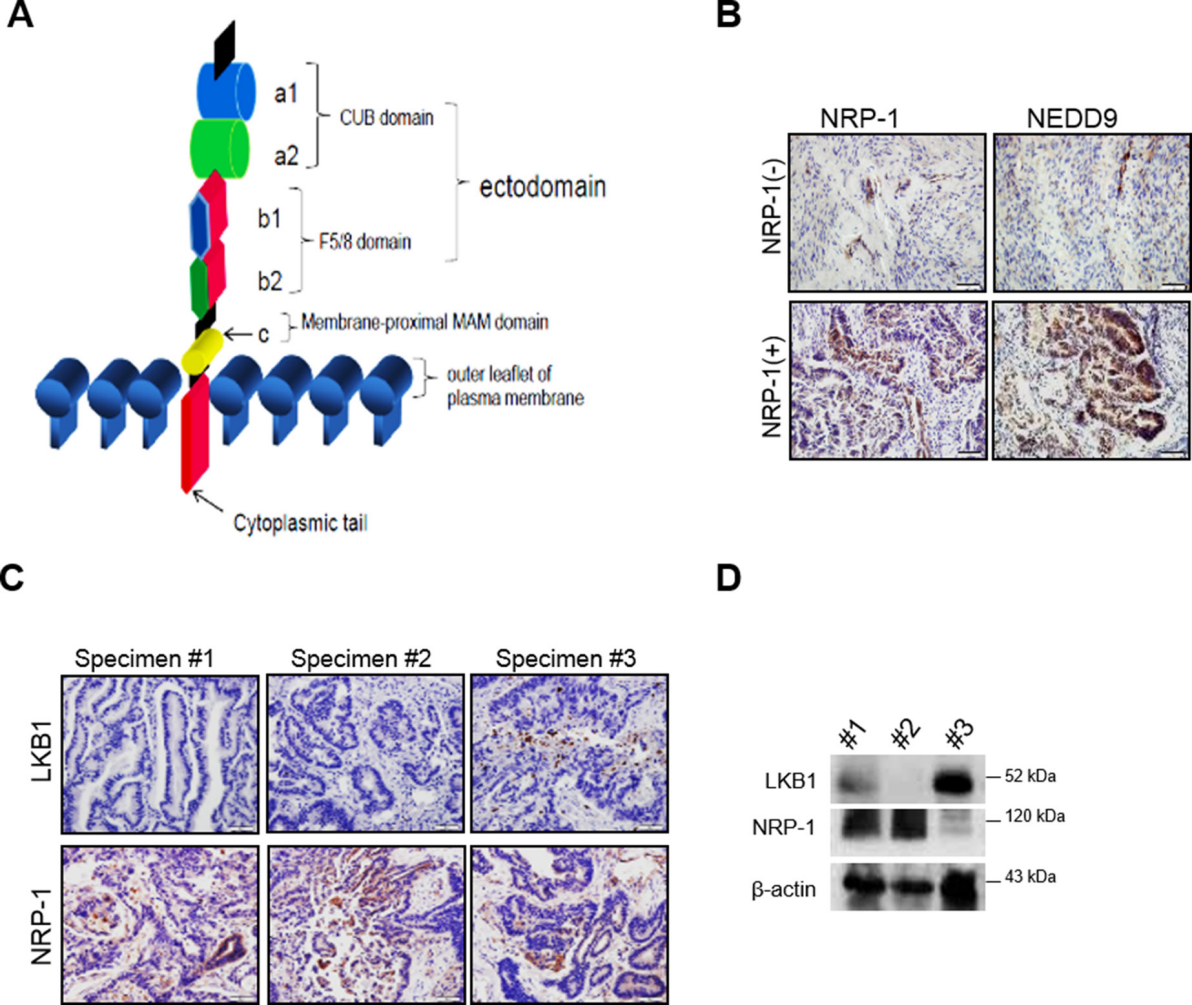
NRP-1 expression positively correlates with metastasis-enhancing NEED9 in human endometrial cancer specimens (**A**) Illustration depicts NRP-1 transmembrane protein structure. (**B**) Representative immunohistochemistry (IHC) data of NRP-1 and NEDD9 expression in NRP-1 positive and negative endometrial cancer specimens. NRP-1 expression positively correlates with NEDD9 gene. (**C** and **D**) Representative IHC and biochemical data demonstrate inverse correlation between LKB1 and NRP-1 expression in endometrial cancer specimens.

Structural arrangement of NRP-1 protein consist of extracellular, transmembrane and intracellular domains (Figure 2A), and has been shown to bind several growth-factors, including HGF [11]. HGF-induced phosphorylation of the c-met receptor drive several processes, including cell proliferation and migration [29, 30]. Furthermore, perturbation of membrane-associated proteins directly impact cell membrane integrity, and hence metastasis. We therefore assessed the expression levels of canonical metastasis-regulating NEDD9 protein in NRP-1 positive and negative specimens [31–34]. NEDD9 is a focal adhesion, scaffolding protein that regulates signaling complexes required for cell attachment, migration and invasion [31, 33]. Immunohistochemical (IHC) analyses of representative specimens demonstrated that NRP-1 positive specimens exhibited strong NEDD9 expression, while NRP-1 negative specimens exhibited negligible NEDD9 expression (Figure 2B). Anti-oncogenic LKB1 functions in opposition to tumor-promoting processes have been widely reported [28, 35, 36]. We previously demonstrated negative correlation between LKB1 and NRP-1 in lung adenocarcinoma [9], and confirmed similar findings in a subset (∼33%) of endometrial tumor specimens (Figure 2C and 2D). Together, the data support a negative association between important cancer targets that possess opposing functions, LKB1 on one hand, and NRP-1/NEDD9 on the other.

### LKB1 depletion enhances metastatic behavior *in vitro* and correlates with increased NRP-1 and NEDD9 expression *in vivo*

**Figure 3:**
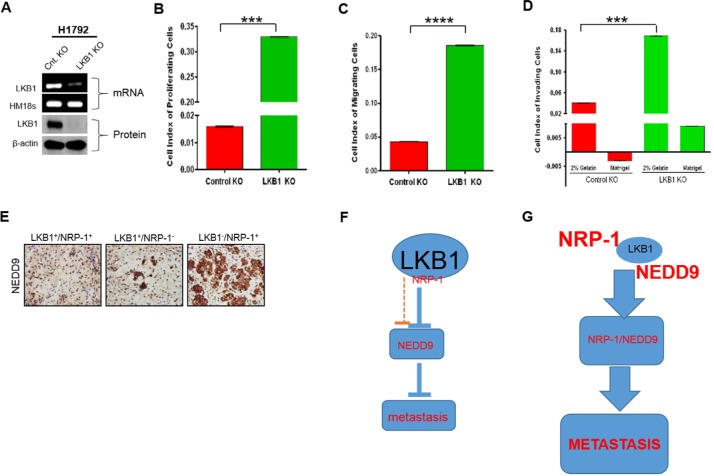
LKB1-deficiency promotes metastatic behavior of cancer cells, and increased NRP-1/NEDD9 expression (**A**) Stable LKB1 (mRNA and protein) knockout (KO) in H1792 cells. (**B**–**D**) LKB1 inhibits tumor-promoting phenotypes. (B) LKB1-expressing H1792 cells demonstrate decreased proliferation compared to LKB1-deficient cells. (***P < 0.001). *P* values correspond to one way ANOVA with Bonferroni's Multiple Comparison Test. *n* = 3. (C and D) LKB1-expressing cells also exhibit decreased migration and invasion across matrigel or a 2% gelatin basement membrane matrix. (**E**) IHC data of mice tumor xenograft confirm increased expression of NEDD9 in NRP-1 + / LKB1- H1792 cell implants. (**F**) In the presence of LKB1, the expressions of NRP-1 and NEDD9 are abrogated, and correlate with decreased metastatic potential of cancer cells. (**G**) LKB1-deficiency correlates with aberrant NRP-1 and NEDD9 expression which enhance the metastatic potential of cancer cells.

Functional consequences of LKB1 deletion was investigated by stably silencing LKB1 expression in NRP-1-positive H1792 metastatic, stage IV lung adenocarcinoma cell line (Figure 3A). We employed a real-time, label-free cell monitoring system to investigate proliferation, migration and invasion. Strong stimulations of these processes were observed in LKB1-shRNA-treated cells compared to control-shRNA-treated cells. LKB1-null cells exhibited an approximately 20-fold increase in proliferative activity compared with LKB1-positive cells (Figure 3B). Measurement of cellular migration over a 36-hour interval demonstrated a significant increase in the migratory potentials of LKB1-null cells compared to LKB1-expressing cells (Figure 3C). Furthermore, assessment of cell invasion into matrigel or gelatin basement membrane matrix demonstrated that LKB1-deficent cells displayed increased invasion (Figure 3D). NEDD9 expression was further evaluated in H1792 xenograft tumors from a previously described mouse model [9]. LKB1 depletion significantly increased tumor growth in association with elevated NRP-1 expression and angiogenesis [9]. IHC analyses of tumors revealed high NEDD9 expression in LKB1-deficient tumors in comparison to LKB1-expressing tumors (Figure 3E). Therefore, our data suggest that LKB1 depletion enhances metastatic behavior of malignant cells *in vitro* (Figure 3B–3D), and correlates with increased expression of NEDD9 metastases-mediating protein *in vivo* (Figure 3E). Figure 3F and 3G depict schematic representation of how the loss or presence of LKB1 impacts NRP-1 and NEDD9 expression, and hence metastasis.

## DISCUSSION

Aberrant NRP-1 expression has been described in human malignancies with links to several tumor phenotypes, including growth, migration, invasion, and angiogenesis [15, 20]. In this study, we gained insight into the positive and negative mediators of pro-metastatic NRP-1 functions in multiple cancers. In endometrial cancer specimens, loss of LKB1 expression inversely correlated with NRP-1 levels. Our previous studies demonstrated that LKB1 promotes NRP-1 degradation and inhibits tumor angiogenesis and growth [9]. In this study, we found that aberrant NRP-1 expression was positively associated with enhanced levels of NEDD9, a protein linked with increased metastases in multiple human cancers [31–34]. Also, we demonstrated that LKB1-depletion was associated with elevated NEDD9 expression in our previously published lung tumor xenograft model in association with increased angiogenesis and tumor growth [9]. This observation is in agreement with recent reports that describe NEDD9 as a target of LKB1 repression in lung cancer [32, 37]. Taken together, these results support a model in which loss of LKB1 allows NRP-1 expression to go unchecked with elevated NEDD9 expression. Furthermore, elevated HGF, a ligand for c-met and NRP-1 that is involved in epithelial-mesenchymal transition and metastatic behavior of cancer cells was significantly associated with NRP-1 expression. The positive association between NRP-1 and HGF expression suggests a positive feedback loop between this receptor and ligand.

Functional roles have been reported for LKB1 with respect to the actin cytoskeleton, as well as modulation of transmembrane receptors [38–41]. In agreement with these observations, our biochemical data demonstrated similar expression pattern of LKB1, Dynamin2 (Dyn2) and NRP-1 in response to treatments with cytochalasin-D (cyto-D), an actin-disrupting agent (Supplementary Figure 1A and 1B). Time-dependent treatments at low cyto-D concentration (1 μg/ml), resulted in decreased expression of all three targets within 30-minutes which may possibly be due to enhanced trafficking or degradation of these targets (Supplementary Figure 1A). However, at higher concentrations (2.5 to 5.0 μg/ml), accumulated protein expression of the targets was observed due to disruption of the actin-membrane network (Supplementary Figure 1B and 1C). The expression pattern of LKB1, Dyn2 and NRP-1 in response to cyto-D treatments suggest these proteins may exist as a complex. Dyn2 is the predominant dynamin in epithelial cells [42], and is intimately associated with actin filament remodeling, as well as endocytosis and membrane trafficking [43]. Depletion of LKB1 and/or Dyn2 expression in hypoxic H1792 cells resulted in the recovery of NRP-1 (Supplementary Figure 1D–1F), suggesting that deletion of LKB1 and/or Dyn2 may abrogate the inhibition of NRP-1. Furthermore, we have observed robust Dyn2 expression across endometrial and lung cancer specimens and cell lines, which appear to be independent of NEDD9. Dyn2 depletion failed to attenuate NEDD9 protein in H1792 cells (data not shown). Because LKB1 kinase activity is necessary for NRP-1 inhibition [9], we speculate that LKB1 may play a role in modifying Dyn2 GTPase activity, as previously observed with Rab7 GTPase [9]. It is possible that LKB1 may alter the ability of Dyn2 to bind cytoskeletal components, or the recruitment of Dyn2 to sites of interest. To this end, it would be imperative to determine whether Dyn2 possess mutations that impact its functionality and GTPase activity within cancer cells. Given the unique structure and pleiotropic co-receptor binding partners of NRP-1, we are currently employing various truncations of the receptor to establish important regulatory/functional domains, and association with NEDD9 protein. Retrospective studies involving NRP-1 expression and further investigations within later-stage endometrial cancer patients are further warranted. In conclusion, findings from this study suggest that aberrant NRP-1 expression may serve as an early biomarker for metastatic endometrial tumors, and potentially influence novel therapies to complement existing interventions.

## MATERIALS AND METHODS

### Cell culture

H1792 lung cancer cell line was purchased from American Type Culture Collection (ATCC; Manassas, VA) and maintained at 37°C in a humidified atmosphere of 5% CO_2_ in an open-air incubator with full medium consisting of RPMI-1640 medium (Sigma, St. Louis, MO) supplemented with 10% (v/v) heat-inactivated fetal bovine serum (FBS), 2 mM glutamine, 100 units/ml penicillin, and 0.1 mg/ml streptomycin.

### Clinical specimens

Matching, de-identified, frozen endometrial cancer and serum specimens were obtained from the OUHSC Stephenson Cancer Center (SCC) Biospecimen Acquisition Core and Bank under an OUHSC IRB approved protocol.

### Transfections and gene silencing

The stable LKB1-deficient H1792 cell line was achieved using lenti-viral shRNAs in pLKO.1-puro plasmid from Santa Cruz Biotechnology (Dallas, TX) and maintained with puromycin selection.

### Western blot analysis and antibodies

Proteins were extracted from the endometrial cancer tissue, electrophoresed into SDS-PAGE gels and transferred to nylon membranes. Membranes were probed with antibodies against NRP-1 (Abcam, San Francisco, CA), LKB1 and β-actin from Santa Cruz Biotechnology. All other antibodies were from Cell Signaling (Beverly, MA). Eleven of the 94 specimens that did not express β-actin as housekeeping controls were eliminated from the analysis. The normalized intensities were given a score of 0 (absent/negligible expression), 1 (moderate expression) or 2 (high expression).

### Multiplex assays of human serum

Serum specimens were evaluated for levels of c-peptide, IL-6, insulin, leptin, MCP-1 (CCL2) and TNFα using a custom-made multiplex kit subset of the MILLIPLEX^®^ MAP Human Metabolic Hormone Magnetic Bead Panel (EMD Millipore), for levels of adipsin and adiponectin, using the Bio-Plex Pro Human Diabetes Adipsin and Adiponectin Assay (BioRad) and for levels of angiopoietin-2, follistatin, G-CSF, HGF, IL-8, leptin, PDGF-BB, PECAM-1 and VEGF using the Bio-Plex Pro^™^ diabetes kit from BIO-RAD Laboratories Inc. (Hercules, CA). Results of specimens with duplicate values that fell above the 15% coefficient of variance limit were removed from the analysis. For each analyte, the average of duplicate values were compared with a standard control curve to derive the serum concentration.

### Migration and invasion assay

Cells were seeded in serum-free medium in upper chambers of uncoated (migration) or 2% gelatin or matrigel-coated (invasion) electrode plates from Roche Applied Science (Indianapolis, IN). The lower chambers contained full media (10% FBS) as a chemoattractant. Cell migration or invasion was monitored in real-time and measured as impedance by the electrode-coated plates using the xCELLgience system (Roche Applied Science).

### Statistical analyses

The intensity scores of NRP-1 expression determined by Western blot were compared with the corresponding cytokine serum concentration using Linear Regression Analysis. The intensity scores for proteins measured on Western blots were compared with each other by Spearman Correlation Analysis. GraphPad 6.0 Software (PRISM) was used for both of these analyses. For both Linear Regression and Spearman Correlation, the r values greater or less than ± 0.2 in association with *p* values < 0.05 were considered statistically significant. The r values greater or less than ± 0.3 associated with *p* values ≥ 0.05 were considered trends. Additional statistical comparisons were performed using a Student's *t*-test or a one-way ANOVA. Values of *p* < 0.05 were considered significant.

### Ethics approval

OUHSC Institutional Review Board for the Protection of Human Subjects approved study employing gynecologic human specimens. (IRB # 3260).

## SUPPLEMENTARY MATERIALS FIGURE



## Abbreviations

NRP-1: neuropilin-1
LKB1: Liver kinase B1
NEDD9: neural precursor cell expressed, developmentally down-regulated 9
Ang-2: angiopoietin-2
G-CSF: granulocyte-colony stimulating factor
HGF: hepatocyte growth factor
IL-8: interleukin-8
PDGF: plateletderived growth factor
PECAM-1: platelet endothelial cell adhesion molecule
VEGF: vascular endothelial growth factor.
