# Supplementary material for: Aberrant NRP-1 expression serves as predicator of metastatic endometrial and lung cancers
Source: Oncotarget. 2015 Dec 20;7(7):7970–8. doi: 10.18632/oncotarget.6699 (PMC4884968; doi:10.18632/oncotarget.6699)
Supplement: Supplementary file 1 [file oncotarget-07-7970-s001.pdf]

Aberrant NRP-1 expression serves as predicator of metastatic endometrial and lung cancers

Supplementary Materials

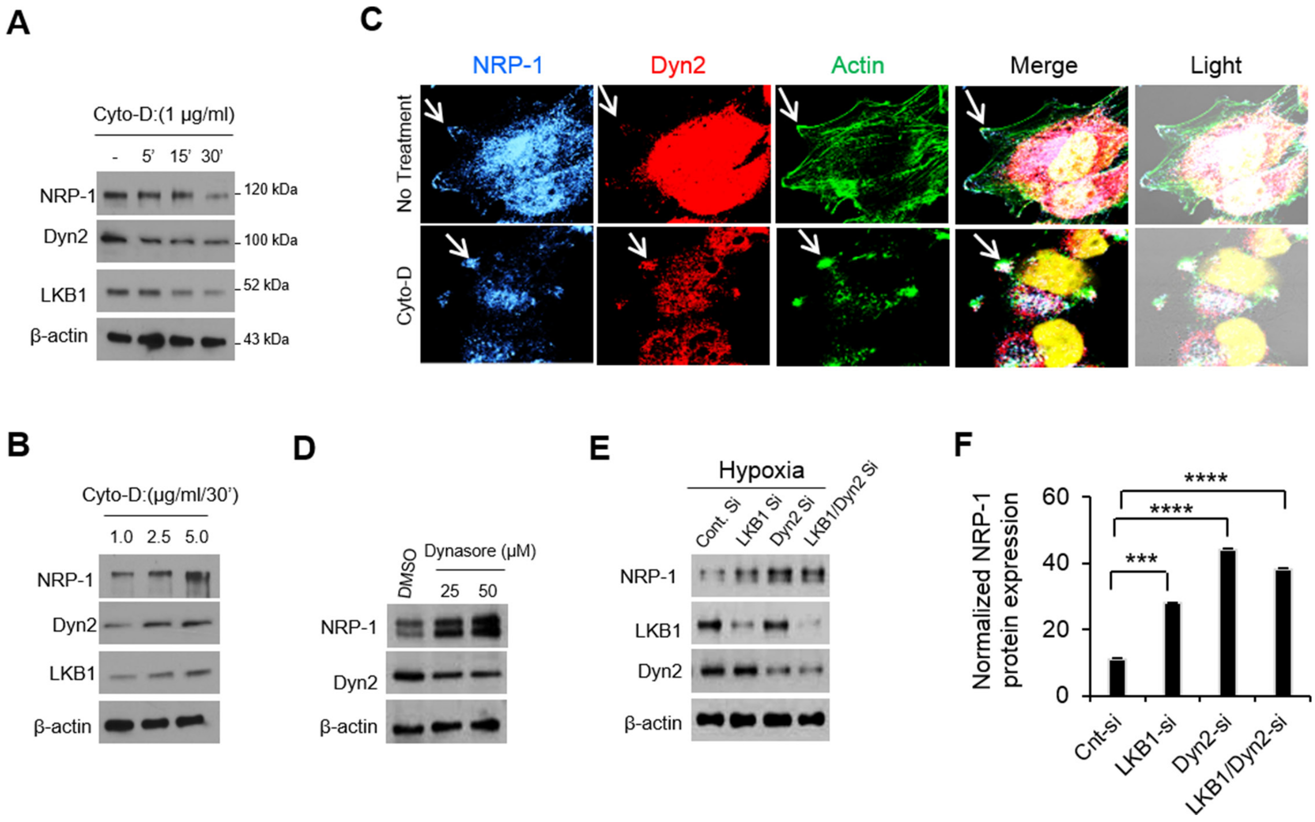

Supplementary Figure S1: LKB1 and/or Dynamin2 expression enhance NRP-1 abrogation. (**A**) Decreased NRP-1 expression upon membrane disruption with Cyto-D (1 μg/ml) in a time-dependent-manner corresponds with reduced LKB1 and Dynamin2 (Dyn2) expression. (**B**) Cytoskeleton disruption following treatments with increased Cyto-D concentrations resulted in the accumulation of NRP-1, LKB1 and Dyn2 proteins. (**C**) Immunofluorescence analysis of NRP-1 (blue), Dyn2 (red) and actin (green) in H1792 cells demonstrate co-localization of NRP-1 and Dyn2 at or near the cell surface (white arrows). Upon membrane disruption with cytochalasin D (Cyto-D), accumulation of actin-Dyn2-NRP-1 in large punctate compartments within the cell was evident (white arrows). (**D**) Dynamin-specific inhibition by dynasore correlated with rescued NRP-1 expression. (E and F) LKBI and/or Dyn2 depletion (siRNA) resulted in the rescue of NRP-1 expression. (**E**) Immunoblots for NRP-1, Dyn2 and LKB1. (**F**) Graph of normalized NRP-1 expression, (****P* < 0.001; *****P* < 0.0001), *P* values correspond to unpaired *t*-test, *n* = 3.
